# Weight Loss Outcomes in a Veterans Affairs Pharmacotherapy-based Weight Management Clinic

**DOI:** 10.1210/jendso/bvae042

**Published:** 2024-03-06

**Authors:** Kevin Ni, Elisa Rogowitz, Abtin K Farahmand, Laura K Kaizer, Jaron Arbet, Christina R Cunningham, Elizabeth A Thomas, David R Saxon

**Affiliations:** Division of Endocrinology, Metabolism, and Diabetes, University of Colorado, Aurora, CO 80045, USA; Division of Endocrinology, Rocky Mountain Regional VA Medical Center, Aurora, CO 80045, USA; Research Service, Rocky Mountain Regional VA Medical Center, Aurora, CO 80045, USA; Division of Endocrinology, Metabolism, and Diabetes, University of Colorado, Aurora, CO 80045, USA; Division of Endocrinology, Rocky Mountain Regional VA Medical Center, Aurora, CO 80045, USA; Division of Endocrinology, Metabolism, and Diabetes, University of Colorado, Aurora, CO 80045, USA; Division of Endocrinology, Rocky Mountain Regional VA Medical Center, Aurora, CO 80045, USA; Department of Biostatistics and Informatics, Colorado School of Public Health, University of Colorado, Aurora, CO 80045, USA; Department of Biostatistics and Informatics, Colorado School of Public Health, University of Colorado, Aurora, CO 80045, USA; Division of Endocrinology, Metabolism, and Diabetes, University of Colorado, Aurora, CO 80045, USA; Division of Endocrinology, Rocky Mountain Regional VA Medical Center, Aurora, CO 80045, USA; Division of Endocrinology, Metabolism, and Diabetes, University of Colorado, Aurora, CO 80045, USA; Division of Endocrinology, Rocky Mountain Regional VA Medical Center, Aurora, CO 80045, USA; Research Service, Rocky Mountain Regional VA Medical Center, Aurora, CO 80045, USA; Division of Endocrinology, Metabolism, and Diabetes, University of Colorado, Aurora, CO 80045, USA; Division of Endocrinology, Rocky Mountain Regional VA Medical Center, Aurora, CO 80045, USA; Research Service, Rocky Mountain Regional VA Medical Center, Aurora, CO 80045, USA

**Keywords:** obesity treatment, weight loss medications, veteran, e-consult

## Abstract

**Context:**

Despite a high prevalence of obesity in the veteran population, antiobesity medications (AOMs) have been underused in the Veterans Health Administration. Real-world reports on outcomes when AOMs have been used in veterans is limited.

**Objective:**

To analyze weight loss outcomes from a local Veterans Health Administration pharmacotherapy-based weight management clinic (WMC).

**Methods:**

This was a retrospective cohort study of veterans enrolled in a local WMC for 15 months from August 2016 through September 2018 and followed through November 2019. Patients were offered 1 of 5 available AOMs based on their comorbidities. Factors associated with weight loss (5% or more weight loss) were assessed.

**Key results:**

A total of 159 patients were seen in a WMC, 149 (93.7%) veterans were prescribed an AOM, and 129 returned for follow-up. Overall, 61/129 (47%) patients achieved 5% or greater weight loss and 28/129 (22%) achieved 10% or greater weight loss within 15 months. Clinically significant weight loss (%) over the first 15 months was achieved with phentermine/topiramate ER (−6.3%) and liraglutide (−7.5%), but not with orlistat (−3.9%) and lorcaserin (−3.6%). Comorbid obstructive sleep apnea was negatively associated with achieving ≥5% weight loss.

**Conclusion:**

Phentermine/topiramate ER and liraglutide were found to be effective AOMs among veterans. Further work is needed to mitigate barriers to AOM initiation given the continued rise in obesity.

Obesity is a national health crisis affecting military veterans. In 2014, obesity and overweight prevalence among more than 5 million veterans receiving primary care in the Veterans Health Administration (VHA) was 41% and 37%, respectively [1]. This prevalence is greater in veterans who receive care at the VHA when compared with those who receive care outside the VHA [2, 3]. Alarmingly, 75% of Iraq and Afghanistan war veterans who use the VHA already have overweight or obesity [4]. To address obesity on a wide scale, the VHA has implemented a national evidence-based behavioral weight management program (MOVE!) with a curriculum focusing on diet, exercise, and behavior change. However, participation rates are low, and weight loss in participants is just 2.2% of body weight after 3 years [5, 6].

One potential way to enhance weight loss success among veterans is the wider adoption of antiobesity pharmacotherapy as an adjunct to behavioral weight management, a strategy that is supported by many professional society guidelines including the VHA/Department of Defense, Endocrine Society, American Heart Association, American College of Cardiology, and The Obesity Society [7-10]. Despite the availability of ant-obesity medications (AOMs) on the VHA's national formulary, utilization of these medications through 2019 was very low with only 1.1% of eligible veterans receiving an AOM prescription within 1 year of MOVE! enrollment [11, 12]. AOMs remain largely underused in the VHA because of various prescription barriers, including restrictive criteria for use [13] and approval and follow-up processes [12]. A recent report from a single VHA site featured a cohort of 43 veterans enrolled during the initial year of their clinic [12] and another pharmacist-run clinic reported 40 patients receiving medications [14], but otherwise descriptions of individual VHA site experiences with AOMs is limited. Despite the recent popularity of semaglutide, a once-weekly glucagon-like peptide-1 receptor agonist, because of its ability to produce a greater magnitude of weight loss than previously available medications [15], its use is limited by medication shortages and national guidelines that restrict it as first-line therapy only for a subpopulation of patients who qualify for AOMs based on standard Food and Drug Administration criteria for AOM use.

In this paper, we report on the clinical outcomes of the earliest years after implementation of a pharmacotherapy-based weight management clinic (WMC) at the Veteran Affairs (VA) Eastern Colorado Health Care System. The goal of this retrospective study is to describe: (1) our model of care for incorporating antiobesity pharmacotherapy into the VA clinical setting, (2) characteristics of patients who received antiobesity pharmacotherapy locally, and (3) the association between initial AOM and outcomes (weight loss and duration of follow up).

## Methods

### Description of Local VHA Weight Management Clinic Structure and Referral Process

The WMC at the VA Eastern Colorado Health Care System was started in August 2016. Partners in the clinic's development included endocrine clinicians (physicians and nurse practitioners), MOVE! dietitians, and a pharmacist all well-versed in VHA national formulary criteria for AOM initiation. Veterans were eligible for the clinic if they participated in MOVE! for at least 3 months and either had a body mass index (BMI) ≥30 kg/m^2^ or a BMI ≥27 kg/m^2^ with at least 1 weight-related comorbidity. Approval for this study was obtained by the institutional review board at the Rocky Mountain Regional Medical Center.

During the 3 years of observation (2016-2019), 5 medications were available for the long-term treatment of obesity in the VHA system: orlistat, lorcaserin, phentermine/topiramate ER (PHEN/TPM), naltrexone/bupropion SR, and liraglutide 3.0 mg. Of note, naltrexone/bupropion SR was nationally approved later (November 2016) and the Food and Drug Administration withdrew lorcaserin approval in February 2020. Liraglutide was a second-line medication based on VHA national criteria (primarily because of its higher cost). Locally, the availability of AOMs was promoted through local MOVE! clinic and primary care providers. Patients attending MOVE! were provided with information explaining the availability of and eligibility criteria for medications.

WMC's referral process initially involved referring all patients to the clinic for a scheduled visit, but because of the large interest in AOMs, an electronic consult (e-consult) process was established to help prescreen patients who were most likely to be eligible for pharmacotherapy based on VA national formulary criteria. In this process, primary care or specialty providers entered an e-consult, which prompted a chart review to determine AOM eligibility. Primary care providers then reviewed the e-consult recommendations with the patient and placed a referral for an in-person consultation if this was still desired by the patient.

At WMC visits, patients eligible for pharmacotherapy underwent a prior authorization drug request reviewed by a pharmacist. Only those patients that met eligibility requirements and had no contraindications to the medication prescribed were approved (Table 1). The VA Criteria for Use required that patients first try at least 1 other AOM (PHEN/TPM, naltrexone/bupropion SR, or orlistat) or have contraindications to these medications before starting liraglutide. In patients with no contraindications to AOMs, selection of an initial AOM was based on shared decision-making and taking into account veteran input regarding the different potential side effects, potential to reach desired weight goals, and patient preferences such as avoiding injections (with liraglutide). Follow-up was defined as having any follow-up visit after the initial WMC visit. Early in the WMC's history, the first follow-up appointment occurred 2 weeks after the initial visit to assess medication tolerance and safety (but not weight loss efficacy), but as the program expanded, first follow-up appointments were scheduled 3 to 4 months after the initial visit. Subsequent visits then generally occurred every 3 to 6 months. Patients were considered lost to follow-up if they had not been seen ≥8 months before the final data collection date of November 30, 2019.

**Table 1.** Veterans Affairs formulary criteria for each weight loss medication

Liraglutide 3.0: Cannot take this if history of suicide attempts or suicidal ideations, alcohol misuse disorder, triglycerides > 1000 mg/dL, or gallstones with intact gallbladder. Can cause nausea, vomiting, diarrhea, constipation, and headache. Risk for pancreatitis, renal failure; contraindicated if patient or family history of MEN2 or medullary thyroid cancer. Lorcaserin: Multiple medications interact with lorcaserin, the primary offenders include metoprolol, tramadol, selective serotonin reuptake inhibitors, serotonin-norepinephrine reuptake inhibitors. Persons aged 65 years and over were not in studies in large-scale trials, given lack of representation; caution is advised when treating older adults with lorcaserin. Can cause hypoglycemia, headache, fatigue. Has also been seen to cause serotonin syndrome, suicidal ideation, heart valve disorder, and bradycardia. Phentermine-topiramate ER: Cannot be used with hyperthyroidism, glaucoma, end-stage renal disease, hepatic dysfunction, cholelithiasis in < 6 months, kidney stones, recurrent major depressive disorder, moderate to severe depression, tramadol, topiramate, monoamine oxidase inhibitors, carbonic anhydrase inhibitors. Naltrexone/bupropion sustained release: Cannot be used in history of uncontrolled hypertension, seizure disorder, bulimia, anorexia, concurrent opioid use, or use of opioids within the past 7-10 days. Excluded for those undergoing abrupt discontinuation of alcohol, benzodiazepines, barbiturates, and antiepileptic drugs. Concurrent use of naltrexone or bupropion. No concurrent use of a CYP2B6 inducer (ritonavir, lopinavir, efavirenz, carbamazepine, phenobarbital, and phenytoin). Orlistat: Cannot be used with history of chronic malabsorption syndrome, chronic diarrhea, cholestasis, or kidney stones.

### Data Collection

Medical record data were obtained for all patients seen in the pharmacotherapy-based WMC whose initial clinic visit occurred between August 1, 2016, and September 20, 2018. Visit data were obtained through November 30, 2019. Data collected included: most recent MOVE! enrollment date and weight; e-consult date and medication recommendations; patient gender, race and ethnicity, age, BMI, self-reported ideal weight; presence of weight-related comorbidities per diagnosis codes or presence of medications or hemoglobin A1c; and weight loss medication. Weights and medication changes were tracked at return visits.

### Outcomes

The primary outcome of this study was weight loss (5% or 10% weight loss from first to last visit within 15 months). Additionally, a secondary aim of this study was to assess factors associated with achieving ≥5% weight loss. Strengthening the report of observational studies in epidemiology-nutritional epidemiology guidelines for reporting observational studies relevant to obesity and dietary research were followed [16].

### Statistical Analysis

Means (SD) or medians (interquartile range) are presented for continuous variables, and n (percent) are presented for categorical variables. We assessed whether there were differences in predictors between those with and without follow up, or those achieving and not achieving 5% and 10% weight loss using 2-sample *t*-tests, Wilcoxon rank-sum tests, chi-square tests, and Fisher exact tests. A generalized linear model (log-binomial) was used to assess whether initial medication was associated with ≥5% weight loss after adjusting for other potential confounders. No additional modeling was done for factors associated with >10% weight loss or follow-up time because of small sample sizes. Significance was assessed at an alpha of 0.05. Because of the exploratory nature of these analyses, *P* values were not adjusted for multiple testing.

## Results

A total of 159 veterans were seen for an initial visit in the WMC. Demographics of all patients (Table 2) who attended the WMC were stratified by having follow-up within 15 months (n = 129) vs those who did not have follow-up within 15 months (n = 30). Those who did not return for a follow-up visit consisted of 20 prescribed AOM at their first WMC visit and 10 veterans who did not receive an AOM for the following reasons: VA formulary contraindications, concern about side effects, and/or uncontrolled psychiatric issues. The 159 veterans who attended their initial WMC visit were, on average, 52.8 (SD, 11.3) years old, 67.3% male, with average weight of 121.6 (25.0) kg, and an average BMI of 40.0 (6.7) kg/m^2^. The median prior-WMC time spent in MOVE! was 204 (interquartile range, 151-306) days, with average weight gain of 0.1 (4.6) %. Prediabetes/type 2 diabetes (median hemoglobin A1c, 5.8%), hypertension, obstructive sleep apnea (OSA), and hyperlipidemia were all common comorbidities. Race/ethnicity was significantly different between those with and without follow-up in the first 15 months (*P* = .037). Approximately 16.7% of those without follow-up were Hispanic/Latino, whereas only 4.7% of those with follow-up were Hispanic/Latino.

**Table 2.** Patient characteristics by WMC follow-up or not

		Follow-up in first 15 months	
Variable	Overall, N = 159*^a^*	No, N = 30*^a^*	Yes, N = 129*^a^*
Age (y)	53 (44, 62)	54 (48, 62)	53 (44, 62)
Sex			
Female	52 (33%)	10 (33%)	42 (33%)
Male	107 (67%)	20 (67%)	87 (67%)
Race/ethnicity			
Non-Hispanic White	109 (69%)	16 (53%)	93 (72%)
Non-Hispanic Black	30 (19%)	9 (30%)	21 (16%)
Hispanic	11 (6.9%)	5 (17%)	6 (4.7%)
Other	3 (1.9%)	0 (0%)	3 (2.3%)
Unknown	6 (3.8%)	0 (0%)	6 (4.7%)
Initial weight (kg)	119 (103, 135)	120 (105, 134)	119 (103, 135)
BMI	39 (35, 45)	40 (35, 42)	38 (35, 45)
BMI categories			
≥27-<30	5 (3.1%)	1 (3.3%)	4 (3.1%)
≥30-<35	36 (23%)	5 (17%)	31 (24%)
≥35-<40	49 (31%)	8 (27%)	41 (32%)
≥40	69 (43%)	16 (53%)	53 (41%)
Hemoglobin A1c	5.80 (5.50, 6.40)	5.80 (5.50, 6.20)	5.75 (5.43, 6.40)
Diabetes			
No	72 (45%)	14 (47%)	58 (45%)
Prediabetes	39 (25%)	8 (27%)	31 (24%)
T2DM	48 (30%)	8 (27%)	40 (31%)
Hypertension	84 (53%)	18 (60%)	66 (51%)
Hyperlipidemia	98 (62%)	18 (60%)	80 (62%)
OSA	46 (29%)	10 (33%)	36 (28%)
Initial AOM			
Orlistat	19 (12%)	3 (10%)	16 (12%)
PHEN/TOP	81 (51%)	12 (40%)	69 (53%)
Liraglutide	40 (25%)	3 (10%)	37 (29%)
Lorcaserin	9 (5.7%)	2 (6.7%)	7 (5.4%)
Did not qualify	10 (6.3%)	10 (33%)	0 (0%)
AOM E-consult	94 (59%)	17 (57%)	77 (60%)
			
Pre-WMC weight loss (%) in MOVE!	0.1 (−2.1, 2.2)	0.1 (−1.5, 1.4)	0.1 (−2.2, 2.2)
Pre-WMC days in MOVE!	204 (151, 306)	193 (150, 352)	204 (152, 302)

Abbreviations: AOM, antiobesity medication; BMI, body mass index; OSA, obstructive sleep apnea; PHEN/TOP, phentermine/topiramate ER; T2DM, type 2 diabetes mellitus; WMC, pharmacotherapy-weight management clinic.

Wilcoxon rank-sum test, Pearson chi-squared test, or Fisher exact test was performed for each variable, all had *P* > .05 except race/ethnicity (*P* < .05) and initial AOM (*P* < .001). Desired weight loss (%) not available for 30 patients (21 with follow-up, 9 without follow-up).

^*a*^Median (interquartile range); n (%).

### Weight Loss Medication Utilization

Initial weight loss medications received (n = 149) were PHEN/TPM (54%), liraglutide (27%), orlistat (13%), and lorcaserin (6%). Patients were allowed the opportunity to switch or restart AOMs as per usual clinical care (Table 3). A total of 122/149 (82%) of patients tried 1 medication, 23/149 (15%) tried 2 medications, and 4/149 tried 3 medications (3%) over the duration of observation. By 15 months after the initial WMC visit, 7/40 patients initially prescribed liraglutide and 10/81 initially prescribed PHEN/TPM had tried a different AOM. Persistence of AOM use among those with follow-up was 97/129 (75%) at ≥5 months, 66/129 (51%) at ≥11 months, and 47/129 (36%) at ≥15 months. Duration of WMC participation with AOM prescription per initial AOM was orlistat (309 days; 95% CI, 168-450; n = 19), PHEN/TPM (387 days; 95% CI, 333-441; n = 81), liraglutide (381 days; 95% CI, 300-463 days; n = 40), and lorcaserin (308 days; 95% CI, 49-568; n = 9).

**Table 3.** Follow-up AOM medication switches and restarts

First-visit AOM	First-visit prescriptions (n)	AOM switch/restart prescriptions (n)				
		Orlistat	PHEN/TOP	Liraglutide	Lorcaserin	Nal/Bup
Liraglutide	40	0	8	3	1	3
Lorcaserin	9	0	1	0	0	0
Orlistat	19	1	2	0	0	0
PHEN/TOP	81	1	1	10	5	0

Abbreviations: AOM, antiobesity medication;; Nal/Bup, naltrexone/bupropion; PHEN/TOP, phentermine/topiramate ER.

AOM switch/restart prescriptions: the number of times a patient for each specific first-visit AOM switched to different AOM or restarted initial AOM were tabulated. Patients who stopped initial AOM at WMC visit or switched to different AOM then restarted initial AOM were tabulated as restarting initial AOM.

### Weight Loss Outcomes

Mean weight loss (within 15 months) among those prescribed AOM and return for follow-up visit was −5.9% (SD, 6.1). Among those with at least 2 follow-up within 15 months after initial medication prescription, a total of 47% (61/129) patients achieved 5% weight loss and 28/129 (22%) achieved 10% or greater weight loss within 15 months. Comparison of patient demographics of those who achieved ≥5% and ≥10% weight loss vs those who did not showed no significant differences in demographics (age, race/ethnicity, BMI, hemoglobin A1c, hypertension, hyperlipidemia). However, there were significant differences in OSA for ≥ 5% weight loss (16% OSA ≥ 5% vs 38% OSA <5%, *P* = .006) and sex for ≥10% weight loss (50% female ≥ 10% vs 28% female <10%, *P* = .026).

**Figure 1.**
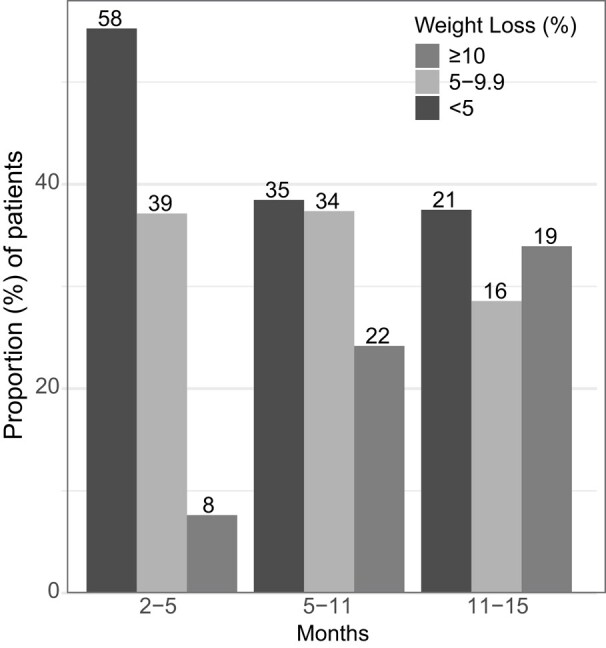
Proportion of patients (%) achieving ≥5% or 10% weight loss who returned for visits during 2 through 5, 5 through 11, or 11 through 15 month follow-up intervals. Bar plot depicting proportions achieving different weight loss targets (<5%, 5%-9.9%, ≥10%) at specified follow-up intervals and labeled with numbers of patients on top.

**Figure 2.**
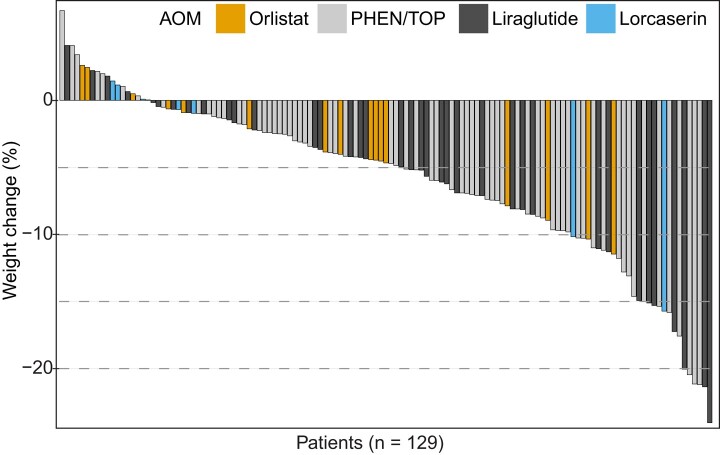
Weight change (%) within the first 15 months among those returning for follow-up visit. Waterfall plot shows individual weight change (%) of patients who returned for follow-up. Each vertical bar depicts one patient, arranged in descending order from maximum weight gain to maximum weight loss, and is colored per initial antiobesity medication prescribed.

**Figure 3.**
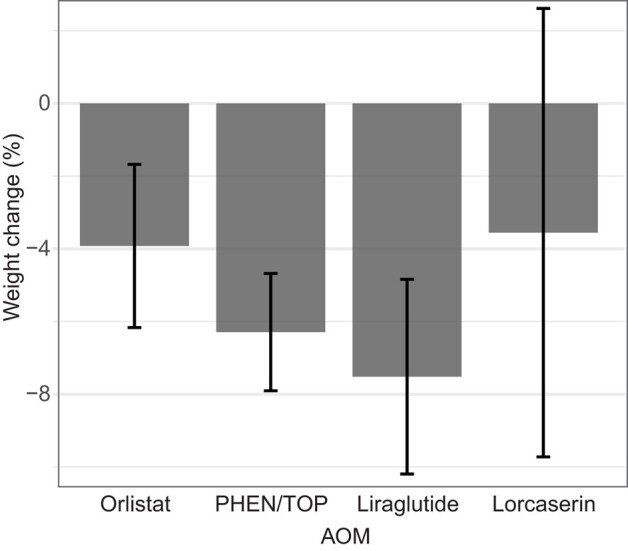
Weight change (%) within the first 15 months per initial antiobesity medication (AOM). Those who switched AOM in first 15 months were excluded. Error bars represent 95% CI. Sample of patients per AOM: orlistat (n = 16), phentermine/topiramate (n = 59), liraglutide (n = 30), and lorcaserin (n = 7).

Of the patients with follow-up at the specified intervals (2-5, 5-11, and 11-15 months), a majority of patients were able to achieve 5% or 10% weight loss (Fig. 1). Among those who returned for follow-up, the distribution of weight loss of individual patients by waterfall plot was notable for a large majority of patients losing weight over the first 15 months (Fig. 2). Excluding those who had a medication switch, statistically significant weight loss (%) over 15 months was achieved with PHEN/TPM (−6.3%, −7.9 to −4.7%, n = 59) and liraglutide (−7.5%, −10.2 to −4.8%, n = 30), but not with orlistat (−3.9; 95% CI, −6.2 to −1.7%; n = 16) and lorcaserin (−3.6%; −9.7 to 2.6%; n = 7), as shown in Fig. 3. Of these patients, the percentage achieving 5% weight loss varied by AOM: PHEN/TPM (n = 31, 53%), liraglutide (n = 18, 60%), orlistat (n = 4, 25%), and lorcaserin (n = 2, 29%).

To assess whether choice of initial AOM was associated with ≥5% weight loss among those with follow up within 15 months, we used a log-binomial generalized linear model, adjusting for OSA and follow up time (in months) (Table 4). After adjusting for OSA and follow-up time, none of the AOMs was associated with more likely to achieve ≥5% weight loss. However, OSA and follow-up time, respectively, were less likely and more likely to achieve ≥5% weight loss. On average, every 1-month increase in follow-up time was associated with 1.06 (95% CI, 1.02-1.12; *P* < .01) times as likely to achieve ≥5% weight loss. Those with OSA, on average, were 0.57 (95% CI, 0.32-0.99; *P* = .047) times as likely to achieve ≥5% weight loss.

**Table 4.** Relative-risk of significant predictors of ≥5% weight loss by multivariable log-binomial analysis

Predictors	Relative risk (95% CI)	*P* value
Follow up time (per month)	1.06 (1.02-1.12)	<.01
Medication		.68
Liraglutide	1.12 (0.79-1.58)	
Lorcaserin	0.89 (0.28-2.82)	
Orlistat	0.66 (0.27-1.59)	
Phentermine/Topiramate	Reference	
Obstructive sleep apnea	0.57 (0.32-0.99)	≤.05

### E-consult Outcomes

Of the 159 patients with initial WMC visit, 94 patients (59%) had an e-consult review for medication eligibility before their in-person appointment. Patients with e-consults spent less time in MOVE! before their first WMC visit than those without e-consults (*P* = .017).

## Discussion

We looked at antiobesity pharmacotherapy effectiveness in an older, predominantly male veteran population. We found that nearly half of participants receiving AOMs lost greater than 5% of their body weight and 22% lost greater than 10%. The mean weight loss in our cohort who received an AOM was −5.9%. We also found that sleep apnea is a potential barrier to AOM-assisted weight loss in veterans. Our results expand on prior real-world studies in the veteran population including a previous study examining weight trends in a national cohort of veterans enriched for those with type 2 diabetes mellitus (82.6%) who received AOM or diabetes medications that showed liraglutide (−7.5% weight loss) and PHEN/TPM (−6.3%) as effective AOM [12]. The magnitude of weight loss achieved with PHEN/TPM in our study was comparable to previous national VHA reports [17]. We focused specifically on a local VHA patient cohort primarily without type 2 diabetes mellitus and report on how we successfully implemented AOM initiation and delivery.

With the increasing incidence of obesity in the VA population [18], there is a critical need to increase AOM initiation and delivery. The VHA's MOVE! program and other nonmedical behavioral weight loss interventions were noted for their modest results (<3% weight loss on average) with difficulty maintaining long-term weight loss [6, 19]. Despite VHA providing AOM at a very affordable cost to veterans, AOMs remained underused for many years until the recent demand for semaglutide [12, 20]. There has been no nationwide integration of AOM delivery within VHA's MOVE though participating in MOVE! or other nonmedical behavioral weight loss intervention has been a requirement for receiving AOM given the importance of healthy dietary/lifestyle behavioral changes for optimal and sustained weight loss. A significant barrier to more widespread AOM use is identifying those who are eligible and providing timely access to pharmacologic treatment. This study is the first to successfully implement e-consults at a local VHA to expedite initiation of AOM.

The VHA presents a unique opportunity to look at AOM outcomes for many important reasons. First, clinical trials of antiobesity pharmacotherapy mostly consist of younger women with few medical problems; therefore, these weight loss outcomes may not be representative of a real-world VA population that is more predominantly older men with a greater number of medical and psychiatric comorbidities. Of note, the percentage of patients (PHEN/TPM 53%, liraglutide 60%) achieving 5% was less than 60% to 70% figures noted by randomized controlled trials [21-23]. Second, the VHA cohort is notable for higher incidence of sleep disturbance, posttraumatic stress disorder (PTSD), and OSA, the effects of which on weight loss and AOM efficacy are not completely known. Third, the VHA patient population is a diverse and marginalized patient population with significant social determinants of health that may contribute to barriers in achieving weight loss, which can be assessed with real-world studies. Last, real-world outcomes from VHA patient cohorts can potentially guide Congressional and Centers for Medicare & Medicaid Services’ review of AOM coverage guidelines [24] given overlapping VHA and Medicare patient populations [25].

This study is based on data from 2016 through 2018, before the approval of once-weekly semaglutide for weight loss. Semaglutide has been shown to result in significantly greater weight loss than any of the medications used in this study [15]. However, despite the potentially greater weight loss benefits with once-weekly semaglutide, this medication is not available first-line to all patients who would benefit from weight loss in the VHA given that current prescribing guidelines for semaglutide require BMI ≥40 or BMI ≥35 with a weight-related comorbidity. In fact, 59/159 (37%) of the patients in our cohort are not eligible for once-weekly semaglutide for weight loss based on current VHA criteria for use. In addition, there are currently national shortages of semaglutide, making it more difficult to obtain the medication even for those patients who would qualify for it. Therefore, VA providers looking to prescribe antiobesity pharmacotherapy need to be versed in the non-semaglutide options highlighted in this study.

We are the first to identify OSA as a potential barrier to weight loss and AOM efficacy in the veteran population. Although weight loss can improve OSA severity [26], OSA diagnosis itself may present a formidable barrier to weight loss. In fact, 1 previous study noted that OSA contributed to decreased benefit of nonmedical weight loss intervention when compared with those without OSA [27]. We speculate that the detrimental effects of OSA and sleep disturbance may be more pronounced in the veteran population given their higher incidence of service-related PTSD and sleep disturbance [28]. Alarmingly, numbers of veterans with OSA, sleep disturbance, and PTSD have significantly increased, with direct links to Iraq and Afghanistan deployment [29, 30], which may present formidable barriers to successful AOM delivery and weight loss in the aging Iraq and Afghanistan veteran population.

Novel strategies are needed to improve the delivery of AOMs within the VHA. One potential strategy to expedite veterans for AOM initiation is through an e-consult pathway that prescreens patients against criteria for use to ensure that patients coming in for discussing, selecting, and starting an AOM are indeed eligible for currently approved AOM given the limited capacity to evaluate everyone for AOM initiation in person [13]. E-consults were successfully piloted here as a strategy to review AOM eligibility before the first in-person WMC visit. E-consult recommendations could also be used to support primary care providers in the VHA with the ability to follow-up and refill AOMs, which is a model frequently adopted when specialty care access is limited. This approach would ensure continued refills, avoid premature AOM termination if patients missed follow-up visits, and allow patients to follow-up closely with their primary care providers who they trust and know well, which emulates primary care-directed weight loss care models outside the VHA.

Several limitations are of note. First, this study has a small sample size, limiting the ability to detect novel predictors of weight loss. Second, those who were referred to and attended WMC likely represent a motivated subset of MOVE! patients very interested in starting AOM and able to engage in WMC clinic visits for AOM fills. Therefore, the AOM buy-in and weight loss results achieved may not apply broadly to all veterans in MOVE! Third, although standardized discussion of benefits and side effects of each medication took place, provider bias can influence shared decision-making regarding medication selection. Fourth, 30% of our cohort had type 2 diabetes, and the potential confounding effect of diabetes medications on weight loss outcomes was not examined.

## Conclusion

Overweight and obesity impact the health of the majority of veterans who receive care in the VHA system. Despite VHA national guidelines supporting the use of AOMs, reports on their effectiveness in the veteran population have been limited. Our results suggest real-world efficacy of PHEN/TPM and liraglutide in veterans. Comorbid OSA was identified as a potential barrier to achieving weight loss and requires further investigation. Strategies to expedite AOM initiation and promote persistent use need to be identified and piloted to address the continued alarming rise of obesity. E-consults may be a powerful tool for fast-tracking AOM initiation.

## Data Availability

Restrictions apply to the availability of some or all data generated or analyzed during this study to preserve patient confidentiality or because they were used under license. The corresponding author will on request detail the restrictions and any conditions under which access to some data may be provided. The data underlying this article will be shared on reasonable request to the corresponding author.

## Abbreviations

AOM: antiobesity medication
BMI: body mass index
OSA: obstructive sleep apnea
PHEN/TOP: phentermine/topiramate ER
PTSD: posttraumatic stress disorder
VA: Veteran Affairs
VHA: Veterans Health Administration
WMC: weight management clinic
